# Comparison of the predictive performance of Cumulative Illness Rating Scale, Charlson Comorbidity Index and COMCOLD Index for moderate-to-severe exacerbations in elderly subjects with chronic obstructive pulmonary disease

**DOI:** 10.1080/07853890.2025.2579789

**Published:** 2025-10-31

**Authors:** Edoardo Pirera, Domenico Di Raimondo, Lucio D’Anna, Riccardo De Rosa, Martina Profita, Sergio Ferrantelli, Davide Paolo Bernasconi, Antonino Tuttolomondo

**Affiliations:** a Internal Medicine and Stroke Care ward, Department of Promoting Health, Maternal-Infant. Excellence and Internal and Specialized Medicine (Promise) G. D’Alessandro, University of Palermo, Palermo, Italy; b Department of Stroke and Neuroscience, Charing Cross Hospital, Imperial College London NHS Healthcare Trust, London, UK; c Bicocca Bioinformatics Biostatistics and Bioimaging Center, School of Medicine and Surgery, University of Milano-Bicocca, Department of Clinical Research and Innovation, ASST Grande Ospedale Metropolitano Niguarda, Milan, Italy

**Keywords:** Comorbidity, COPD, acute exacerbation of COPD, Cumulative Illness Rating Scale, CIRS, Charlson Comorbidity Index, COMCOLD

## Abstract

**Background and objective:**

Chronic Obstructive Pulmonary Disease (COPD) is frequently associated with multiple comorbidities that influence clinical outcomes. This study aimed to compare the predictive performance of the Cumulative Illness Rating Scale (CIRS) with the Charlson Comorbidity Index (CCI) and COMCOLD Index for moderate-to-severe COPD exacerbations.

**Materials and methods:**

We conducted a prospective observational study involving 200 COPD patients followed for 52 weeks. CIRS indices (Total Score, Severity Index, Comorbidity Index), CCI, and COMCOLD were calculated at baseline. The primary outcome was time-to-first moderate-to-severe exacerbation. Cox regression analyses and time-dependent receiver operating characteristic curves were used to assess prognostic performance at 12, 24, and 52 weeks.

**Results:**

During follow-up, 66 patients (33%) experienced at least one moderate-to-severe exacerbation. All CIRS indices demonstrated significant correlations with respiratory parameters and symptom burden. In crude models, CIRS indices were significantly associated with exacerbation risk (CIRS-TS: HR 1.11, 95%CI 1.06–1.16; CIRS-SI: HR 1.16, 95%CI 1.09–1.23; CIRS-CI: HR 1.37, 95%CI 1.20–1.56; all *p* < 0.001), maintaining significance after adjustment for clinical covariates. CIRS indices demonstrated superior discriminative performance compared to CCI and COMCOLD, with CIRS-SI achieving the highest time-dependent AUC values across all timepoints (0.704, 0.679, and 0.778 at 12, 24, and 52 weeks, respectively).

**Conclusion:**

CIRS provides superior prognostic accuracy compared to established comorbidity indices in identifying COPD patients at increased risk of exacerbations. These findings highlight the clinical relevance of incorporating a comprehensive, severity-weighted comorbidity assessment in COPD management, supporting the concept of COPD as a complex, multisystem disorder requiring an integrated approach to care.

## KEY MESSAGES

In elderly patients with COPD, CIRS provided superior prognostic accuracy for moderate-to-severe exacerbations compared with the Charlson Comorbidity Index and COMCOLD;

The prognostic advantage of CIRS likely derives from its comprehensive, severity-weighted assessment of multimorbidity across multiple organ systems;

Incorporating multidimensional comorbidity evaluation, such as CIRS, into clinical practice may improve risk stratification and support more personalized COPD management.

## 1. Introduction

Chronic Obstructive Pulmonary Disease (COPD) is a progressive respiratory disorder characterized by persistent airflow limitation that is not fully reversible [1] caused by small airways disease and parenchymal destruction, most commonly due to prolonged exposure to harmful particles or gases, especially tobacco smoke. COPD remains a significant global public health challenge, as it is the third leading cause of death globally and is a substantial contributor to morbidity, mortality, and healthcare system utilization [1,2]. Projections indicate that increasing smoking rates in low- and middle-income countries, combined with population aging in high-income nations, may drive the annual number of deaths attributable to COPD and related conditions to surpass 5.4 million by 2060 [3].

COPD is increasingly recognized as a multisystemic disorder, frequently associated with multiple comorbid conditions that extend beyond its direct effects on the respiratory system. This relationship is not merely coincidental but rather driven by shared risk factors, such as smoking, aging, and chronic low-grade systemic inflammation [4]. The interaction between COPD and its comorbid conditions has significant implications for prognosis, therapeutic strategies, and healthcare utilization [5]. Over time, patients often develop malnutrition, experience a progressive decline in functional capacity, and report a diminished quality of life [6].

Given that, an evaluation that extends beyond airflow limitation—typically assessed using forced expiratory volume in one second (FEV_1_)—is essential [7,8]. Accurately quantifying comorbidity burden is critical for optimizing patient management and improving clinical outcomes. In this context, a multidimensional assessment of multimorbidity may enhance risk stratification, allowing for the identification of high-risk patients who may benefit from more intensive surveillance and individualized therapeutic approaches. Several indices have been developed to evaluate comorbidity burden and predict outcomes in patients with COPD. For instance, the Charlson Comorbidity Index [9] (CCI), originally designed for the general population, has been widely applied in COPD research and clinical practice. However, because it was not specifically tailored to respiratory disease, it may underestimate the prognostic role of conditions more directly relevant to COPD. The “Comorbidities in Chronic Obstructive Lung Disease” (COMCOLD) Index [10] was specifically developed in COPD populations to address this gap by focusing on a selected set of comorbidities that have a well-established impact on quality of life and prognosis. In contrast, the Cumulative Illness Rating Scale [11] (CIRS) provides a broader, severity-weighted assessment of multimorbidity across multiple organ systems, making it particularly relevant in geriatric and chronic disease populations.

We hypothesized that a multidimensional approach incorporating a severity-weighted comorbidity index such as the CIRS could improve risk stratification by more accurately distinguishing individuals at increased risk of moderate-to-severe exacerbations, as compared to other established indices, namely CCI and COMCOLD Index—which assess a narrow spectrum of comorbidities.

The aim of our study is to compare the prognostic accuracy of CIRS with CCI and COMCOLD, in estimating short- and medium-term exacerbation risk. In this study, the comparison between CIRS, CCI, and COMCOLD was conducted across three distinct domains: (1) correlation of each index with COPD severity and dyspnea burden; (2) hazard ratios (HR) for time to first moderate-to-severe COPD exacerbation; (3) prognostic accuracy reported as time-dependent Area Under the Curve (timeAUC) values for exacerbation events during follow-up.

## 2. Methods

### 2.1. Study design and population

This was a prospective and observational study involving subject with COPD enrolled at “Internal Medicine and Stroke Care” ward and “COPD and Cardiovascular Risk” Outpatient Unit of the “Policlinico Paolo Giaccone” of the University of Palermo from 01/09/2021 to 01/09/2024. This is a preliminary analysis of the ongoing MACH (Multidimensional Approach for COPD and High Complexity) trial, which is registered on the ClinicalTrials.gov platform (NCT04986332) and has been approved by our institutional review board (Comitato Etico Palermo 1, approval ref. no. 04/2021). MACH is a three-year, prospective study primarily designed to evaluate the impact of a multidimensional intervention targeting both COPD and major comorbidities on clinical and laboratory outcomes. In contrast, this interim analysis focuses on prognostic evaluation of comorbidity indices and exacerbation risk. The study was conducted in accordance with the ethical standards of the relevant institutional and national committees, as well as the 1964 Declaration of Helsinki. Written Informed consent was obtained from each participant enrolled in the study.

Participants were considered eligible for this study if they fulfilled all of the following criteria: (1) Male or female aged ≥ 65 years; (2) confirmed COPD diagnosis based on the Global Initiative for Chronic Obstructive Lung Disease (GOLD) [1]. (3) Able to provide a signed and dated informed consent form. Patients with history of current asthma were excluded.

Each participant was followed up for 52 weeks, with scheduled visits at 12, 24, and 52 weeks to collect information about the occurrence of moderate or severe exacerbations.

### 2.2. COPD assessment and outcomes

At the time of enrolment, each participant was assessed for dyspnea, using the modified Medical Research Council (mMRC) scale and the COPD Assessment Test (CAT), and spirometry was performed using the POXY FX desktop spirometer (COSMED Srl, Rome, Italy). Forced Vital Capacity (FVC) maneuvers were performed in accordance with the most recent international guidelines [12]. The Global Lung Initiative (GLI) reference equations for spirometry were used [13]. According to the GOLD guidelines [1], COPD severity is classified into: GOLD 1 with FEV_1_ ≥ 80% predicted; GOLD 2 with FEV_1_ 50–79% predicted; GOLD 3 with FEV_1_ 30–49% predicted; and GOLD 4 with FEV_1_ < 30% predicted. Additionally, we classified patients into three GOLD categories: Category A, representing patients with few symptoms (mMRC < 2 or CAT < 10) and low exacerbation risk (0–1 exacerbations without hospitalization in the previous year); Category B, including patients with more symptoms (mMRC ≥ 2 or CAT≥ 10) but still low exacerbation risk; and Category E, including patients with high exacerbation risk (≥ 2 moderate exacerbations or ≥ 1 exacerbation leading to hospitalization in the previous year) regardless of symptom burden. The primary outcome of the study was a composite of a moderate or severe acute exacerbation at 52–weeks of follow-up. According to the latest international recommendations [1], a moderate exacerbation was defined as a worsening of the respiratory symptoms requiring treatment with a short-acting bronchodilator, oral corticosteroids or antibiotics. Conversely, a severe exacerbation was defined as a worsening in respiratory symptoms leading to hospitalization or an emergency department visit.

### 2.3. Prognostic indices

For the purpose of this study, the following indices were calculated:

1. CIRS: Originally introduced by Linn et al., the CIRS score provides a detailed evaluation of both the presence and severity of diseases across 14 organ systems using a 0–4 rating scale [14]. Later, Salvi et al. refined this methodology by standardizing the scoring criteria with explicit examples provided in the CIRS manual [11]. Three indices have been developed based on the CIRS: the total score (CIRS-TS), which represents the sum of the scores across all 14 organ systems; the severity index (CIRS-SI), calculated as the mean score of the first 13 categories, excluding psychiatric/behavioral disorders; and the comorbidity index (CIRS-CI), which quantifies the number of categories—excluding psychiatric/behavioral disorders—where a score of 3 or higher is recorded [11]. The CIRS score is recognized as a valuable indicator of health status and has been shown to predict both 18-month mortality and rehospitalization in hospitalized elderly patients [11,15]. Furthermore, CIRS indices are associated with the risk of acute exacerbations of COPD and they correlate with the severity of respiratory symptoms and lung function [16];
2. CCI: This is one of the most commonly used tools to assess the impact of comorbidities on overall survival [17]. Originally developed by Charlson et al. in 1987, it assigns weighted scores to 19 medical conditions based on their relative risk of mortality. A higher total CCI score indicates a greater burden of comorbidity and an increased risk of adverse outcomes. Although the CCI is widely employed in COPD patients [18,19], a noted limitation is its failure to account for several common conditions frequently associated with COPD, such as hypertension, atrial fibrillation, anxiety, pulmonary fibrosis, and depression [20];
3. COMCOLD: Frei et al. developed an index specifically in COPD cohorts to assess the overall impact of comorbidities on patient-reported health status, using the feeling thermometer—a visual analogue scale ranging from 0 (dead) to 100 (perfect health). This index does not include respiratory variables, assigning a score (0–19) based on five conditions: depression, anxiety, peripheral artery disease, cerebrovascular disease, and symptomatic heart disease [10];

The same trained investigator administered the scores to all enrolled participants. The detailed characteristics of all prognostic indices utilized in this analysis—COMCOLD, CCI and CIRS—including their evaluated domains, parameters, scoring criteria, and clinical relevance, are summarized in Supplementary Table S1.

### 2.4. Statistical analysis

Qualitative variables were reported as absolute frequencies and percentages, while quantitative variables were summarized as mean and standard deviation (SD) for normally distributed data or as median and interquartile range (IQR) for non-normally distributed data. After 52-week follow-up, the cohort was divided into two groups based on primary outcome. Baseline clinical characteristics were compared between groups using Student’s t-test for continuous variables with normal distribution, the Wilcoxon rank-sum test for those with non-normal distribution, and the chi-square test for categorical variables. Spearman’s nonparametric test was used to assess correlations between baseline measures of CIRS indices, CCI, COMCOLD index and mMRC, CAT score, FEV_1_ and FVC. Cox regression analysis was performed to evaluate the association between CIRS Indices, CCI, COMCOLD Index and the time to first moderate-to-severe exacerbation. For each index, we fitted both univariate (crude) and multivariate models accounting for age, sex, mMRC scale, and GOLD Class (Class 1 as reference) and GOLD Category (Category A as reference). Additionally, we fitted a separate “respiratory model” incorporating these covariates without comorbidity indices. In the survival analysis, death secondary to an acute COPD exacerbation was considered as an outcome event, whereas deaths from other causes were censored at the time of occurrence. To evaluate the prognostic accuracy of each model, both crude and adjusted, we conducted time-dependent receiver operating characteristic (timeROC) analyses, computing and comparing the timeAUC at 12, 24, and 52 weeks using the “timeROC” R package [21] which estimates AUCs through inverse probability of censoring weighting, as described by Blanche et al. [22]. All models were internally validated through 10-fold cross-validation, partitioning the dataset into ten equal segments and iteratively training on nine while testing on the remaining subset. A two-tailed p value < 0.05 was considered significant and 95% confidence interval (95%CI) was reported. The statistical analysis was conducted with R 4.4.1 (Foundation for Statistical Computing, Vienna, Austria).

## 3. Results

A total of 200 individuals with COPD were included in the final analysis (Supplementary Figure S1). During the follow-up, six participants died for COPD exacerbation. The baseline characteristics of the study population including age, sex, BMI, smoking status and COPD severity in terms of airflow limitation, exacerbation status and dyspnea are presented in Table 1. The median age of the overall population was 74 years (IQR: 69–80), with 64% being male. The majority of patients were classified as GOLD category E (69%), while 8% and 23% were in category A and B, respectively. During the follow-up period, 66 patients (33%) experienced at least one moderate or severe exacerbation. Patients who experienced exacerbations had lower lung function parameters, with a significantly reduced FEV_1_, lower FEV_1_% predicted and worse mMRC and CAT scores. Moreover, individuals with moderate or severe exacerbations had significantly greater comorbidity burdens, as reflected by higher scores in CIRS-TS, CIRS-SI, CIRS-CI (*p* < 0.001), CCI (*p* = 0.02). No significant difference was observed in the COMCOLD Index between groups (*p* = 0.512).

**Table 1.** Baseline characteristics of the study population stratified by exacerbation status during follow-up.

Variables	Overall population	Moderate or severe exacerbation during follow-up	No moderate or severe exacerbation during follow-up	*p*-value
Number	*n* = 200	*n* = 66	*n* = 134	N/A
Age, median years (IQR)	74 (69–80)	74 (69–80)	75 (69–79)	0.809
Men, n (%)	128 (64)	45 (68.2)	83 (61.9)	0.387
Body Mass Index, median kg/m^2^ (IQR)	27.9 (24.2–31.2)	28.1 (24.5–31.5)	27.7 (24.2–31.1)	0.653
Active smoker, n (%)	82 (41)	24 (36.3)	58 (43.2)	0.349
GOLD category, n (%)	A: 16 (8) B: 46 (23) E: 138 (69)	A: 1 (1.52) B: 13 (19.7) E: 52 (78.8)	A: 15 (11.2) B: 33 (24.6) E: 86 (64.2)	0.03
FEV_1_, median Lt/sec (IQR)	1.43 (1.06–1.85)	1.24 (0.84–1.76)	1.52 (1.16–1.87)	0.036
FEV_1_, % predicted	58.5 ± 19.0	53.5 ± 19.7	60.2 ± 18.3	0.02
FVC, Lt/sec	2.37 ± 0.74	2.17 ± 0.66	2.47 ± 0.76	0.025
FVC, % predicted	72 ± 17.9	68.5 ± 15.9	76.7 ± 18.4	0.011
Inhaled Therapy, n (%)	LAMA: 34 (17)	LAMA: 5 (7.6)	LAMA: 29 (21.7)	0.154
	LABA/LAMA: 69 (34)	LABA/LAMA: 25 (37.9)	LABA/LAMA: 44 (32.8)	
	LABA/ICS: 24 (12)	LABA/ICS: 9 (13.6)	LABA/ICS: 15 (11.2)	
	LAMA/LABA/ICS: 73 (36.50)	LAMA/LABA/ICS: 27 (40.9)	LAMA/LABA/ICS: 46 (34.3)	
mMRC	2 ± 1.03	2.89 ± 0.47	2.08 ± 0.97	<0.001
CAT	16 ± 7.7	19.5 ± 7.1	14.3 ± 7.4	<0.001
CIRS-TS	17.7 ± 6.1	20.4 ± 5.8	16.4 ± 5.8	<0.001
CIRS-SI	1.4 ± 0.45	1.5 ± 0.45	1.2 ± 0.43	<0.001
CIRS-CI, median (IQR)	3 (2–4)	4 (3–5)	3 (1–3)	<0.001
Charlson Comorbidity Index	6 ± 2.2	6.8 ± 2.3	6 ± 2.3	0.02
COMCOLD Index, median (IQR)	3 (3–6)	3 (3–6)	3 (3–6)	0.512

Data are presented as mean ± standard deviation, unless otherwise specified. Exacerbation status refers to the occurrence or absence of events during the 52-week follow-up. Abbreviations: GOLD: Global Initiative for Chronic Obstructive Lung Disease; FEV_1_: Forced Expiratory Volume in one second; FVC: Forced Vital Capacity; mMRC: modified Medical Research Council; CAT: COPD Assessment Test; CIRS-TS: Cumulative Illness Rating Scale Total Score; CIRS-SI: Cumulative Illness Rating Scale Severity Index; CIRS-CI: Cumulative Illness Rating Scale Comorbidity Index; LAMA: long-acting muscarinic antagonist; LABA: long‐acting beta‐agonists; ICS: Inhaled Corticosteroid.

### 3.1. Correlation among CIRS Indices, COMCOLD, Charlson Comorbidity Index and the COPD severity and dyspnea burden

Correlation analysis between comorbidity indices and respiratory variables is shown in Table 2. CIRS-TS, CIRS-SI, and CIRS-CI demonstrated significant positive correlations with the CCI (*r* = 0.640, *r* = 0.627, and *r* = 0.620, respectively; *p* < 0.001) and the COMCOLD Index (*r* = 0.512, *r* = 0.476, and *r* = 0.428, respectively; *p* < 0.001). CIRS indices also showed significant positive correlation with mMRC (r range: 0.443 to 0.453; *p* < 0.001) and CAT score (r range: 0.346 to 0.364; *p* < 0.001). Interestingly, CIRS indices exhibited significant negative correlations with lung function parameters, most notably with FVC% predicted (r ranging from −0.311 to −0.317; all *p* < 0.001) and to a lesser extent with FEV_1_. While the CCI showed similar correlation patterns with symptom (mMRC: *r* = 0.350, *p* < 0.001; CAT score: *r* = 0.313, *p* < 0.001), its correlations with lung function parameters were mostly non statistically significant, except for FVC in liters (r=-0.210, *p* < 0.05). The COMCOLD index showed similar results to the CCI.

**Table 2.** CIRS vs Charlson Comorbidity Index vs COMCOLD Index: Correlation analysis with baseline respiratory parameters.

	Clinical variables						Comorbidity indices	
	mMRC	CAT Score	FEV_1_ (% predicted)	FEV_1_ (Lt/sec)	FVC (% predicted)	FVC (Lt)	Charlson Comorbidity Index	COMCOLD Index
CIRS-TS	0.453***	0.364***	−0.230**	−0.212*	−0.317***	−0.271**	0.640***	0.512***
CIRS-SI	0.443***	0.356***	−0.232***	−0.217*	−0.316***	−0.276**	0.627***	0.476***
CIRS-CI	0.450***	0.346***	−0.215**	−0.235**	−0.311***	−0.265**	0.620***	0.428***
Charlson Comorbidity Index	0.350***	0.313***	−0.120	−0.165	−0.159	−0.210*	/	0.506***
COMCOLD Index	0.242***	0.206**	−0.053	−0.111	−0.109	−0.175*	0.506***	/

*p < 0.05; **p < 0.01; ***p < 0.001.

Data are presented as Spearman’s correlation coefficient, ρ (rho). All indices and respiratory parameters refer to baseline measurements. Abbreviations: FEV_1_: Forced Expiratory Volume in one second; FVC: Forced Vital Capacity; mMRC: modified Medical Research Council; CAT: COPD Assessment Test; CIRS-TS: Cumulative Illness Rating Scale Total Score; CIRS-SI: Cumulative Illness Rating Scale Severity Index; CIRS-CI: Cumulative Illness Rating Scale Comorbidity Index.

### 3.2. Crude and adjusted Cox regression analysis for time to first moderate-to-severe COPD exacerbation

Table 3 presents the results of Cox regression analyses for the association between the three comorbidity indices considered and COPD exacerbation risk. Cox regression analysis showed that each unit increase in CIRS-TS was associated with a 11% increased risk of COPD exacerbation in the crude model (crude HR = 1.11, 95%CI: 1.06–1.16, *p* < 0.0001) and an 8% increased risk in the adjusted model (adjusted HR = 1.08, 95%CI: 1.03–1.14, *p* < 0.0001). Similar results were observed for a 0.1 increase of CIRS-SI (crude HR = 1.16, 95%CI: 1.09–1.23; adjusted HR = 1.13, 95%CI: 1.05–1.21) and 1-point increase of CIRS-CI (crude HR = 1.37, 95%CI: 1.20–1.56; adjusted HR = 1.33, 95%CI: 1.13–1.56). The CCI showed a significant association in the crude model only (crude HR = 1.14, 95%CI: 1.03–1.27, *p* < 0.05), while the COMCOLD Index was not associated with exacerbation risk in either model. Both crude and adjusted Cox regression models are fully reported in Supplementary Table S2.

**Table 3.** Comparison of discriminative performance of CIRS indices vs CCI and COMCOLD for moderate-to-severe COPD exacerbations at 12, 24 and 52 weeks.

	CIRS-TS	CIRS-SI†	CIRS-CI	COMCOLD	Charlson Comorbidity Index
Crude model	1.11 (1.06–1.16)***	1.16 (1.09–1.23)***	1.37 (1.20–1.57)***	1.02 (0.94–1.10)	1.15 (1.03–1.27)*
Adjusted model	1.09 (1.03–1.14)**	1.13 (1.05–1.21)***	1.33 (1.13–1.57)***	0.98 (0.90–1.07)	1.06 (0.94–1.19)

*p < 0.05; **p < 0.01; ***p < 0.001. **^†^**(0.1-point increase).

Data are reported as hazard ratio and 95%CI. In all models, results represent the risk associated with a one-point increase in each prognostic index, unless otherwise specified. Adjusted model included Age, Sex, mMRC, GOLD Class (Class 1 as reference) and GOLD Category (Category A as reference). Abbreviations: CIRS-TS: Cumulative Illness Rating Scale Total Score; CIRS-SI: Cumulative Illness Rating Scale Severity Index; CIRS-CI: Cumulative Illness Rating Scale Comorbidity Index.

### 3.3. Results of time-dependent AUC analysis of CIRS indices, CCI and COMCOLD Index

Results of time-dependent area under the curve (time-AUC) analysis are presented in Table 4. In the crude models, CIRS indices consistently showed better prognostic performance compared to COMCOLD and CCI. Specifically, at 12 weeks, CIRS indices showed the highest discriminative performance (timeAUC of CIRS-TS: 0.700; CIRS-SI: 0.704; CIRS-CI: 0.683) compared to the CCI and COMCOLD Index (0.565 and 0.637, respectively). This pattern was observed across all time points, with CIRS indices (e.g. CIRS-SI at 24 weeks: 0.741; at 52 weeks: 0.679), while CCI (at 24 weeks: 0.650; at 52 weeks: 0.586) and COMCOLD performed poorly (at 24 weeks: 0. 552; at 52 weeks: 0.522). CIRS indices showed higher AUC values than COMCOLD and, to a lesser extent, CCI, with some differences reaching statistical significance, although the 95%CI of the estimates overlapped across indices at several timepoints.

**Table 4.** Comparison of prognostic performance of CIRS indices vs CCI and COMCOLD for moderate-to-severe COPD exacerbations at 12, 24 and 52 weeks.

Timepoint	Model	CIRS-TS	CIRS-SI	CIRS-CI	COMCOLD	Charlson Comorbidity Index	Respiratory model
12 weeks	Crude	0.700 (0.586–0.813)	0.704 (0.590–0.818)	0.683 (0.583–0.782)	0.565 (0.471–0.658)	0.637 (0.534–0.739)	0.772 (0.666–0.878)
	Adjusted	0.786 (0.678–0.893)	0.787 (0.681–0.894)	0.777 (0.670–0.883)	0.773 (0.667–0.879)	0.774 (0.673–0.876)	
24 weeks	Crude	0.724 (0.634–0.814)	0.741 (0.651–0.831)	0.716 (0.635–0.798)	0.552 (0.467–0.638)	0.650 (0.562–0.737)	0.718 (0.619–0.819)
	Adjusted	0.767 (0.680–0.855)	0.778 (0.692–0.864)	0.764 (0.676–0.851)	0.719 (0.620–0.818)	0.720 (0.626–0.815)	
52 weeks	Crude	0.677 (0.595–0.813)	0.679 (0.597–0.761)	0.672 (0.594–0.750)	0.522 (0.443–0.601)	0.586 (0.500–0.671)	0.724 (0.645–0.803)
	Adjusted	0.750 (0.677–0.823)	0.754 (0.681–0.827)	0.752 (0.680–0.824)	0.729 (0.651–0.808)	0.719 (0.641–0.797)	

Data are presented as time-dependent AUC (95%CI) estimated using inverse probability of censoring weighting. Abbreviations: CIRS-TS: Cumulative Illness Rating Scale Total Score; CIRS-SI: Cumulative Illness Rating Scale Severity Index; CIRS-CI: Cumulative Illness Rating Scale Comorbidity Index.

After adjustment for confounding variables, all indices showed improved predictive accuracy. The CIRS-SI maintained the highest timeAUC values across all time points (0.787, 0.778, and 0.754 at 12, 24, and 52 weeks, respectively). The respiratory model showed comparable discriminative performance to the adjusted models, with timeAUC values of 0.772, 0.718, and 0.724 at 12, 24, and 52 weeks, respectively. However, these timeAUC remained consistently lower than those of multivariate models incorporating CIRS indices. Comparisons of adjusted models and the respiratory model showed no significant differences between CIRS indices and either COMCOLD or CCI at most timepoints, with the only exception of CIRS-SI vs the respiratory model at 24 weeks (*p* = 0.049), nevertheless 95%CI overlapped across indices. Pairwise comparison of timeAUC is fully reported in Supplementary Table S3. Cross-validation revealed minimal overfitting across all models, with CIRS indices maintaining higher prognostic accuracy compared to COMCOLD and CCI. Adjusted models demonstrated higher timeAUC values, reflecting meaningful contribution from respiratory variables, while COMCOLD showed greatest instability under validation in crude models. Cross-validation analysis is reported in Supplementary Table S4.

## 4. Discussion

In this study, we evaluated the association of CIRS indices with the risk of moderate-to-severe exacerbations during a 52-weeks follow-up and their prognostic performance compared to CCI and COMCOLD, indices already validated in COPD patients, using time-dependent AUC analyses. The main findings of our study were that the CIRS indices are significantly correlated with CCI and COMCOLD and are associated with COPD exacerbation risk. Specifically, our Cox regression analysis showed that each unit increase in CIRS-TS was associated with an 11% increased risk of COPD exacerbation in the crude model and an 8% increased risk in the adjusted model, the CCI showed a significant association only in the crude model, while the COMCOLD index was not associated with exacerbation risk in either model. The CIRS indices consistently showed higher prognostic performance compared to COMCOLD and CCI, a finding that remained robust after adjustment for confounding and COPD severity variables. Indeed, inclusion of these variables improved the discriminatory ability of all models. This shows that the scores perform better when evaluated together with clinical information. Although CIRS indices achieved higher AUC values compared with COMCOLD and with CCI, the overlap of 95%CI indicates that these differences, while statistically significant in some comparisons, should be interpreted with caution.

The higher prognostic performance of the CIRS may be attributed to its ability to comprehensively capture the burden of comorbidity, rather than simply enumerating the diseases associated with COPD in the individual patient. Unlike CCI, which omits several common conditions frequently coexisting with COPD—such as hypertension, atrial fibrillation, anxiety, pulmonary fibrosis, and depression—CIRS systematically evaluates 14 organ systems, offering a more detailed assessment of comorbidity burden. More importantly, CIRS quantifies not only the presence but also the severity of comorbidities, which may represent a critical determinant of prognosis. The severity of coexisting conditions likely reflects the extent of systemic inflammation, oxidative stress, and physiological dysregulation, all of which can significantly influence disease progression, treatment response, and exacerbation risk in COPD [23,24].

Several authors highlight that the coexistence of multiple diseases in patients with COPD has a significant clinical and prognostic relevance, with a higher incidence of multimorbidity than observed in subjects without COPD [25]. Despite the well-documented associations between COPD and its comorbidities, current mechanistic explanations remain insufficient to fully elucidate the underlying pathobiological connections between these conditions [26]. Indeed, COPD and its associated comorbidities are still classified as separate clinical entities rather than being recognized as different manifestations of common underlying processes. However, growing evidence suggests that these associations may arise from shared pathobiological mechanisms [27].

In recent years, there has been renewed interest in exploring the association between COPD and multimorbidity, culminating in the proposal of a syndemic framework: the emerging concept of syndemics refers to the concurrent or sequential clustering of two or more epidemics within a population, characterized by biological interactions that amplify their overall effects [28]. In this context, the intricate relationship between COPD and cardiovascular (CV) diseases represents a well-documented syndemic, specifically termed “cardiopulmonary risks” in contemporary literature [29]. This hypothesis is supported by several recent studies: (1) a meta-analysis by Chen et al. demonstrated that CV diseases are nearly two to five times more prevalent in COPD patients compared with those without COPD [30]; (2) our group recently demonstrated through a meta-analysis of 16 observational studies including >1.8 million patients that the risk of CV events after an acute exacerbation follows a precise temporal trajectory. Specifically, the risk of CV events is greatest within 30 days after a COPD exacerbation gradually diminishes over time, remaining higher for up to one year, especially for acute coronary syndrome and heart failure [31]; (3) Moreover, CV comorbidity significantly modifies clinical outcomes in COPD patients, while COPD similarly exacerbates CV prognosis. Extensive epidemiological evidence demonstrates that COPD confers substantial excess mortality and morbidity among patients with established CV diseases and viceversa [32–34]; (4) While various maintenance therapies exist for COPD to improve airflow limitation and reduce exacerbations, only inhaled triple therapy—a combination of long-acting muscarinic antagonist (LAMA), a long-acting beta_2_ agonist (LABA) and inhaled corticosteroid (ICS)—has demonstrated a reduction in all-cause mortality [35,36]. This finding may be partially explained by evidence indicating that as many as two-thirds of patients with COPD ultimately die from non-pulmonary causes, with CV disease being the leading contributor [37,38]. A more comprehensive assessment of disease severity across multiple organ systems using a score such as the CIRS, may be useful for the clinician to support the “cardiopulmonary” syndemic concept in daily practice. Given that CV conditions represent the most prevalent and prognostically significant comorbidities in COPD patients [26], the detailed assessment of CV disease severity—rather than mere presence—of CIRS indices likely contributes substantially to its prognostic advantage. Beyond our findings, recent advances in pharmacovigilance for cardiovascular adverse events and innovative machine-learning approaches to cardiovascular risk prediction may be applied to COPD populations, offering novel opportunities to refine the evaluation of cardiovascular comorbidities and to develop more accurate prognostic tools [39,40].

Recent literature, along with the findings of this study, underscores a crucial reality: the management of COPD cannot be confined to a single specialty. Our study emphasizes the critical importance of a multidisciplinary assessment for all patients with COPD, a necessity that becomes even more imperative during episodes of acute exacerbation. Such an integrated approach is essential to ensure comprehensive management, encompassing not only overt CV conditions but also the identification and treatment of underlying CV risk factors. Effective COPD care must be tailored to the multifaceted nature of the disease, necessitating collaborative, multidisciplinary strategies addressing both the evolution of the respiratory syndrome and comorbidities simultaneously, as they are mutually influencing each other.

This study presents several methodological and practical limitations that should be acknowledged. First, the relatively modest sample size of 200 patients, with only 66 experiencing the primary outcome, may have limited the statistical power and the stability of multivariable models, potentially affecting the reliability of the effect estimates reported in our study. Second, the study population consisted entirely of elderly patients, largely falling within GOLD Category E. This restricts the generalizability of the findings to younger individuals or those with milder disease. Third, the single-center design may limit external validity, as patient characteristics, healthcare resources, and management practices may vary considerably across institutions. Fourth, the 52-week follow-up period, while sufficient to capture short- and medium-term outcomes, may be relatively limited to fully assess long-term exacerbation risk and the sustained prognostic performance of the indices over time. Additionally, our analysis did not consider potential confounders such as vaccination status or pulmonary rehabilitation, which may influence exacerbation risk and could have affected the observed associations. While 10-fold internal cross-validation showed minimal overfitting, external validation in independent COPD cohorts would be valuable to confirm reproducibility and support broader clinical applicability. In this context, future multicenter prospective validation studies are warranted to verify these findings and to determine whether the observed improvements in prognostic performance translate into measurable clinical benefits. Such investigations are essential to determine whether tailored interventions, informed by this comprehensive comorbidity assessment, can effectively modify the integrated cardiopulmonary risk profile and ultimately improve the long-term prognosis of individuals with COPD.

## 5. Conclusion

This study indicates that the CIRS score is a more effective tool for predicting the risk of future exacerbations in elderly COPD patients with a higher risk of moderate-to-severe COPD exacerbations compared to established indices through its more comprehensive assessment of the comorbidity burden.

Our findings highlight the clinical and prognostic relevance of routinely incorporate in clinical practice a comprehensive, severity-weighted comorbidity assessment in COPD management. Given the complexity of COPD and its strong interconnection with CV and other systemic comorbidities, the results also reinforce the need for a multidisciplinary and integrated approach in the care of these patients. Further validation in larger studies and broader clinical settings is necessary to confirm the generalizability and clinical applicability of our conclusions.

## Supplementary Material

Supplementary materials Pirera_R1.docx

Legend to the tables.docx

STROBE Checklist_Pirera.doc

## Data Availability

The data that support the findings of this study are available from the corresponding author, upon reasonable request.
