# Supplementary material for: Comparison of the predictive performance of Cumulative Illness Rating Scale, Charlson Comorbidity Index and COMCOLD Index for moderate-to-severe exacerbations in elderly subjects with chronic obstructive pulmonary disease
Source: Ann Med. 2025 Oct 31;57(1):2579789. doi: 10.1080/07853890.2025.2579789 (PMC12581735; doi:10.1080/07853890.2025.2579789)
Supplement: Supplementary materials Pirera_R1.docx [file IANN_A_2579789_SM6512.docx]

**Supplementary materials of:** “COMPARISON OF THE PREDICTIVE PERFORMANCE OF CUMULATIVE ILLNESS RATING SCALE, CHARLSON COMORBIDITY INDEX AND COMCOLD INDEX FOR MODERATE-TO-SEVERE EXACERBATIONS IN ELDERLY SUBJECTS WITH CHRONIC OBSTRUCTIVE PULMONARY DISEASE”

**Authors:** Edoardo Pirera, MD^a, §^; Domenico Di Raimondo, MD, PhD^a, § *,^; Lucio D’Anna, MD, PhD^b^, Riccardo De Rosa, MD^a^; Martina Profita, MD^a^; Sergio Ferrantelli, MD^a^; Davide Paolo Bernasconi, PhD^c^; Antonino Tuttolomondo, MD, PhD^a^;

^a^ Internal Medicine and Stroke Care ward, Department of Promoting Health, Maternal-Infant. Excellence and Internal and Specialized Medicine (Promise) G. D'Alessandro, University of Palermo; Palermo (Italy);

^b^ Department of Stroke and Neuroscience, Charing Cross Hospital, Imperial College London NHS Healthcare Trust, London, United Kingdom (UK);

^c^ Bicocca Bioinformatics Biostatistics and Bioimaging Center, School of Medicine and Surgery, University of Milano-Bicocca, 20854 Vedano al Lambro, Italy; Department of Clinical Research and Innovation, ASST Grande Ospedale Metropolitano Niguarda, 20126 Milan, Italy;

^*^ Corresponding author;

# **Supplementary Table 1.** Comparative Table of Indices for “Comorbidity Burden”

| **Index** | **Evaluated Domains** | **Parameters** | **Scoring System (points)** | **Score Range** | **Notes** |
| --- | --- | --- | --- | --- | --- |
| **COMCOLD Index** | Precence of comorbidities | - Depression; - Anxiety; - Peripheral arterial disease; - Cerebrovascular disease; - Symptomatic heart disease; | - Depression: 6 points; - Anxiety: 4 points; - Peripheral arterial disease: 3 points; - Cerebrovascular disease: 3 points; - Symptomatic heart disease: 3 points; | 0-19 | Specifically evaluates impact of comorbidities on quality of life. Cerebrovascular disease includes stroke or transient ischemic attack. Heart disease includes coronary heart disease and/or heart failure. |
| **Charlson Comorbidity Index** | Presence of comorbidities;  Severity assessment only for “Liver disease”, “Diabetes Mellitus” and “Solid Tumor” | - 19 clinical conditions with variable scores; | - Conditions with 1 point: myocardial infarction, congestive heart failure, peripheral vascular disease, cerebrovascular disease, dementia, chronic pulmonary disease, rheumatologic disease, peptic ulcer disease, mild liver disease, diabetes; - Conditions with 2 points: hemiplegia, moderate-to-severe renal disease, diabetes with chronic complications, malignancy without metastases, leukemia, lymphoma; - Conditions with 3 points: moderate or severe liver disease; - Conditions with 6 points: metastatic solid tumor, AIDS; | 0-37 | Generic comorbidity index not specific to COPD. Widely used in various clinical contexts. Evaluates comorbidities' impact on mortality. |
| **Cumulative Illness Rating Scale** | **Presence and severity of comorbidities across 14 organ systems** | - 14 categories of organ systems; - Each category rated on severity from 0 to 4; | - Total Score: sum of all category scores; - Severity Index: mean score of the first 13 categories, excluding psychiatric/behavioral disorders; - Comorbidity Index: number of categories with score ≥2, excluding psychiatric/behavioral disorders; | Total Score: 0-56;  Severity Index: 0-4;  Comorbidity Index: 0-13; | Provides more detailed assessment of comorbidities. Considers severity of each condition. Not specific to COPD but allows for comprehensive evaluation of multimorbidity. |

**Supplementary Figure 1 – Study Flowchart**

**
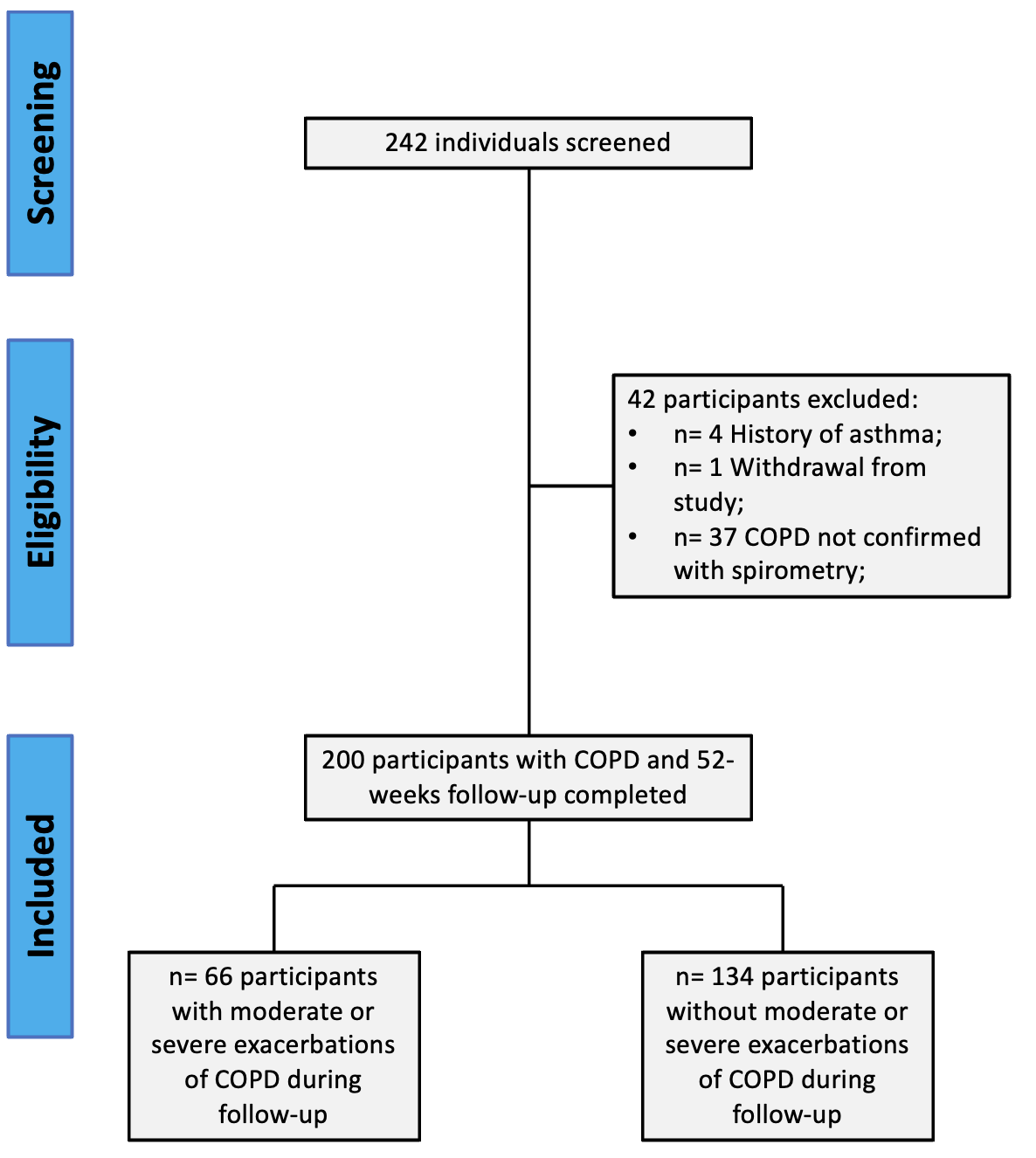
**

**Supplementary Table 2.** Results of multivariable Cox regression analysis.

|  | **Adjusted Models** | | | | | |
| --- | --- | --- | --- | --- | --- | --- |
| **Variable** | **CIRS-TS** | **CIRS-SI** | **CIRS-CI** | **COMCOLD**  **Index** | **Charlson**  **Comorbidity**  **Index** | **Respiratory** |
| Age | 0.95 (0.91-0.99)** | 0.95 (0.91-0.99)** | 0.94 (0.91-0.98)** | 0.97 (0.93-1.01) |  | 0.96 (0.93-1.00) |
| Sex | 0.74 (0.43-1.28) | 0.74 (0.42-1.28) | 0.69 (0.40-1.21) | 0.81 (0.47-1.40) | 0.87 (0.50-1.49) | 0.82 (0.48-1.41) |
| mMRC | 1.94 (1.38-2.73)*** | 1.91 (1.36-2.69)*** | 1.96 (1.39-2.75)*** | 2.31 (1.65-3.25)*** | 2.15 (1.52-3.04)*** | 2.28 (1.64-3.19)*** |
| GOLD  Class | 1.08 (0.50-2.31) | 1.07 (0.50-2.29) | 1.02 (0.48-2.18) | 1.03 (0.48-2.20) | 0.93 (0.44-1.97) | 1.05 (0.50-2.23) |
| GOLD  Category | 1.10 (0.36-3.34) | 1.10 (0.36-3.33) | 1.19 (0.39-3.62) | 1.25 (0.41-3.82) | 1.06 (0.35-3.21) | 1.26 (0.41-3.82) |
| CIRS-TS | 1.09 (1.03-1.14)** | / | / | / | / | / |
| CIRS-SI | / | 1.13 (1.05-1.21)*** | / | / | / | / |
| CIRS-CI | / | / | 1.33 (1.13-1.57)*** | / | / | / |
| COMCOLD  Index | / | / | / | 0.98 (0.90-1.07) | / | / |
| Charlson  Comorbidity  Index | / | / | / | / | 1.06 (0.94-1.19) | / |
| * p<0.05; ** p<0.01; *** p<0.001 | | | | | | |

**Supplementary Table 3.** Comparison of time-dependent AUC between CIRS, Charlson Comorbidity Index and COMCOLD Index at different time points.

| **Comparison** | **12 weeks** | **24 weeks** | **52 weeks** |
| --- | --- | --- | --- |
| CIRS-TS vs COMCOLD (Crude) | 0.006** | <0.001*** | <0.001*** |
| CIRS-SI vs COMCOLD (Crude) | 0.005** | <0.001*** | <0.001*** |
| CIRS-CI vs COMCOLD (Crude) | 0.018* | <0.001*** | <0.001*** |
| CIRS-TS vs Charlson (Crude) | 0.148 | 0.064 | 0.010* |
| CIRS-SI vs Charlson (Crude) | 0.130 | 0.033* | 0.011* |
| CIRS-CI vs Charlson (Crude) | 0.269 | 0.086 | 0.013* |
| CIRS-TS vs COMCOLD (Adjusted) | 0.700 | 0.124 | 0.408 |
| CIRS-SI vs COMCOLD (Adjusted) | 0.678 | 0.074 | 0.348 |
| CIRS-CI vs COMCOLD (Adjusted) | 0.905 | 0.158 | 0.338 |
| CIRS-TS vs Charlson (Adjusted) | 0.711 | 0.145 | 0.215 |
| CIRS-SI vs Charlson (Adjusted) | 0.684 | 0.091 | 0.184 |
| CIRS-CI vs Charlson (Adjusted) | 0.927 | 0.186 | 0.175 |
| CIRS-TS vs Respiratory (Adjusted) | 0.620 | 0.083 | 0.249 |
| CIRS-SI vs Respiratory (Adjusted) | 0.602 | 0.049* | 0.215 |
| CIRS-CI vs Respiratory (Adjusted) | 0.847 | 0.112 | 0.189 |

**Supplementary Table 4** – Results of 10k-fold cross validation for the timeAUC of CIRS Indices vs CCI and COMCOLD for moderate-to-severe COPD exacerbations at 12, 24 and 52 weeks.

| **Timepoint** | **Model** | **CIRS-TS** | **CIRS-SI** | **CIRS-CI** | **COMCOLD** | **Charlson**  **Comorbidity Index** | **Respiratory**  **Model** |
| --- | --- | --- | --- | --- | --- | --- | --- |
| 12 weeks | Crude | 0.694  (0.580–0.807) | 0.698  (0.585–0.811) | 0.671  (0.566–0.776) | 0.413  (0.301–0.526) | 0.620  (0.515–0.725) | 0.753  (0.647–0.859) |
|  | Adjusted | 0.758  (0.649–0.867) | 0.761  (0.652–0.870) | 0.754  (0.646–0.862) | 0.744  (0.634–0.854) | 0.756  (0.652–0.860) |  |
| 24 weeks | Crude | 0.711  (0.620–0.802) | 0.729  (0.638–0.820) | 0.689  (0.602–0.775) | 0.373  (0.282–0.465) | 0.613  (0.521–0.704) | 0.686  (0.584–0.788) |
|  | Adjusted | 0.730  (0.638–0.822) | 0.739  (0.648–0.830) | 0.718  (0.625–0.811) | 0.683  (0.580–0.786) | 0.690  (0.590–0.789) |  |
| 52 weeks | Crude | 0.668  (0.584–0.751) | 0.672  (0.589–0.756) | 0.650  (0.568–0.733) | 0.411  (0.324–0.497) | 0.556  (0.468–0.644) | 0.684  (0.601–0.768) |
|  | Adjusted | 0.704  (0.625–0.783) | 0.704  (0.625–0.783) | 0.692  (0.612–0.771) | 0.680  (0.597–0.764) | 0.667  (0.583–0.752) |  |

Data are presented as time-dependent AUC (95%CI) estimated using inverse probability of censoring weighting. Abbreviations: CIRS-TS: Cumulative Illness Rating Scale Total Score; CIRS-SI: Cumulative Illness Rating Scale Severity Index; CIRS-CI: Cumulative Illness Rating Scale Comorbidity Index;
