# Supplementary material for: Comparison of the predictive performance of Cumulative Illness Rating Scale, Charlson Comorbidity Index and COMCOLD Index for moderate-to-severe exacerbations in elderly subjects with chronic obstructive pulmonary disease
Source: Ann Med. 2025 Oct 31;57(1):2579789. doi: 10.1080/07853890.2025.2579789 (PMC12581735; doi:10.1080/07853890.2025.2579789)
Supplement: Legend to the tables.docx [file IANN_A_2579789_SM6511.docx]

**COMPARISON OF THE PREDICTIVE PERFORMANCE OF CUMULATIVE ILLNESS RATING SCALE, CHARLSON COMORBIDITY INDEX AND COMCOLD INDEX FOR MODERATE-TO-SEVERE EXACERBATIONS IN ELDERLY SUBJECTS WITH CHRONIC OBSTRUCTIVE PULMONARY DISEASE**

**Legend to the tables:**

# **Table 1 – Baseline Characteristics of the Study Population Stratified by Exacerbation Status During Follow-up.**

Data are presented as mean ± standard deviation, unless otherwise specified. Exacerbation status refers to the occurrence or absence of events during the 52-week follow-up.

Abbreviations: GOLD: Global Initiative for Chronic Obstructive Lung Disease; FEV_1_: Forced Expiratory Volume in one second; FVC: Forced Vital Capacity; mMRC: modified Medical Research Council; CAT: COPD Assessment Test; CIRS-TS: Cumulative Illness Rating Scale Total Score; CIRS-SI: Cumulative Illness Rating Scale Severity Index; CIRS-CI: Cumulative Illness Rating Scale Comorbidity Index; LAMA: long-acting muscarinic antagonist; LABA: long‐acting beta‐agonists; ICS: Inhaled Corticosteroid;

# **Table 2 – CIRS vs Charlson Comorbidity Index vs COMCOLD Index: Correlation analysis with baseline respiratory parameters.**

Data are presented as Spearman's correlation coefficient, ρ (rho). All indices and respiratory parameters refer to baseline measurements. Abbreviations: FEV1: Forced Expiratory Volume in one second; FVC: Forced Vital Capacity; mMRC: modified Medical Research Council; CAT: COPD Assessment Test; CIRS-TS: Cumulative Illness Rating Scale Total Score; CIRS-SI: Cumulative Illness Rating Scale Severity Index; CIRS-CI: Cumulative Illness Rating Scale Comorbidity Index;

**Table 3 – Comparison of discriminative performance of CIRS Indices vs CCI and COMCOLD for moderate-to-severe COPD exacerbations at 12, 24 and 52 weeks.**

Data are reported as Hazard Ratio and 95%CI. In all models, results represent the risk associated with a one-point increase in each prognostic index, unless otherwise specified. Adjusted model included Age, Sex, mMRC, GOLD Class (Class 1 as reference) and GOLD Category (Category A as reference). Abbreviations: CIRS-TS: Cumulative Illness Rating Scale Total Score; CIRS-SI: Cumulative Illness Rating Scale Severity Index; CIRS-CI: Cumulative Illness Rating Scale Comorbidity Index;

**Table 4 – Comparison of prognostic performance of CIRS Indices vs CCI and COMCOLD for moderate-to-severe COPD exacerbations at 12, 24 and 52 weeks.**

Data are presented as time-dependent AUC (95%CI) estimated using inverse probability of censoring weighting. Abbreviations: CIRS-TS: Cumulative Illness Rating Scale Total Score; CIRS-SI: Cumulative Illness Rating Scale Severity Index; CIRS-CI: Cumulative Illness Rating Scale Comorbidity Index;
